# Molecular networks discriminating mouse bladder responses to intravesical bacillus Calmette-Guerin (BCG), LPS, and TNF-α

**DOI:** 10.1186/1471-2172-9-4

**Published:** 2008-02-11

**Authors:** Marcia R Saban, Michael A O'Donnell, Robert E Hurst, Xue-Ru Wu, Cindy Simpson, Igor Dozmorov, Carole Davis, Ricardo Saban

**Affiliations:** 1 College of Medicine, Department of Physiology, Oklahoma University Health Sciences Center (OUHSC), Oklahoma City, OK 73104, USA; 2 Department of Urology, University of Iowa, UI Hospitals and Clinics, Iowa City, Iowa 52242-1089, USA; 3 Departments of Urology, Biochemistry, and Molecular Biology, The University Oklahoma Health Sciences Center, Oklahoma City, OK 73104, USA; 4 Department of Urology, New York University, School of Medicine, New York, NY 10016, USA; 5 Oklahoma Medical Research Foundation (OMRF), Arthritis and Immunology Research Program, Microarray/Euk. Genomics Core Facility, Oklahoma City, Oklahoma 73104, USA

## Abstract

**Background:**

Despite being a mainstay for treating superficial bladder carcinoma and a promising agent for interstitial cystitis, the precise mechanism of Bacillus Calmette-Guerin (BCG) remains poorly understood. It is particularly unclear whether BCG is capable of altering gene expression in the bladder target organ beyond its well-recognized pro-inflammatory effects and how this relates to its therapeutic efficacy. The objective of this study was to determine differentially expressed genes in the mouse bladder following chronic intravesical BCG therapy and to compare the results to non-specific pro inflammatory stimuli (LPS and TNF-α). For this purpose, C57BL/6 female mice received four weekly instillations of BCG, LPS, or TNF-α. Seven days after the last instillation, the urothelium along with the submucosa was removed from detrusor muscle and the RNA was extracted from both layers for cDNA array experiments. Microarray results were normalized by a robust regression analysis and only genes with an expression above a conditional threshold of 0.001 (3SD above background) were selected for analysis. Next, genes presenting a 3-fold ratio in regard to the control group were entered in Ingenuity Pathway Analysis (IPA) for a comparative analysis in order to determine genes specifically regulated by BCG, TNF-α, and LPS. In addition, the transcriptome was precipitated with an antibody against RNA polymerase II and real-time polymerase chain reaction assay (Q-PCR) was used to confirm some of the BCG-specific transcripts.

**Results:**

Molecular networks of treatment-specific genes generated several hypotheses regarding the mode of action of BCG. BCG-specific genes involved small GTPases and BCG-specific networks overlapped with the following canonical signaling pathways: axonal guidance, B cell receptor, aryl hydrocarbon receptor, IL-6, PPAR, Wnt/β-catenin, and cAMP. In addition, a specific detrusor network expressed a high degree of overlap with the development of the lymphatic system. Interestingly, TNF-α-specific networks overlapped with the following canonical signaling pathways: PPAR, death receptor, and apoptosis. Finally, LPS-specific networks overlapped with the LPS/IL-1 mediated inhibition of RXR. Because NF-kappaB occupied a central position in several networks, we further determined whether this transcription factor was part of the responses to BCG. Electrophoretic mobility shift assays confirmed the participation of NF-kappaB in the mouse bladder responses to BCG. In addition, BCG treatment of a human urothelial cancer cell line (J82) also increased the binding activity of NF-kappaB, as determined by precipitation of the chromatin by a NF-kappaB-p65 antibody and Q-PCR of genes bearing a NF-kappaB consensus sequence. Next, we tested the hypothesis of whether small GTPases such as LRG-47 are involved in the uptake of BCG by the bladder urothelium.

**Conclusion:**

As expected, BCG treatment induces the transcription of genes belonging to common pro-inflammatory networks. However, BCG also induces unique genes belonging to molecular networks involved in axonal guidance and lymphatic system development within the bladder target organ. In addition, NF-kappaB seems to play a predominant role in the bladder responses to BCG therapy. Finally, in intact urothelium, BCG-GFP internalizes in LRG-47-positive vesicles.

These results provide a molecular framework for the further study of the involvement of immune and nervous systems in the bladder responses to BCG therapy.

## Background

Intravesical Bacillus Calmette-Guerin (BCG) is best known as the most effective agent for the treatment of high-grade superficial bladder cancer [1-3]. In this context, BCG is used to reduce both the recurrence rate of bladder tumor and to diminish the risk of its progression [1,2]. As an adjunct to transurethral resection, BCG is the treatment of choice for urothelial carcinoma in-situ (CIS) and is commonly used for recurrent or multi-focal Ta and high grade T1 bladder lesions [4,5]. BCG also has been tested as a promising option for treatment of interstitial cystitis [6].

It is not clear how BCG alters the course of cystitis or cancer progression. One theory is that intravesical BCG corrects an aberrant immune imbalance in the bladder, leading to long-term symptomatic improvement [1].

Recently, the susceptibility to BCG was correlated with polymorphisms of the human NRAMP1 gene [7], providing interesting insights into the complexity of the genomics of BCG immunotherapy [8].

That BCG causes an extensive local inflammatory reaction in the bladder wall is well acknowledged [9]. Of this, the massive appearance of cytokines in the urine of BCG-treated cancer patients stands out [9]. Activated lymphocytes and macrophages are the most likely sources of these cytokines, but at present other cellular sources such as urothelial cells cannot be ruled out [9]. BCG is internalized and processed by neutrophils [10], professional antigen-presenting cells and urothelial tumor cells, resulting in altered gene expression and secretion of particular cytokines [9]. It was suggested that the effectiveness of BCG treatment is determined by two processes: an inflammatory one, followed by a delayed type of hypersensitivity response [11]. Others proposed three distinct phases in the immune response to BCG. In phase 1, BCG adheres to the urothelium via interaction between the bacterial antigen 85 complex and fibronectin [5,12] and urothelial cells. In addition to fibronectin, it has been suggested that toll-like receptors (TLRs) -2 and -4, present in immune cells, mediate BCG-induced immune responses [13-15]. Once internalized, BCG is processed both by professional antigen-presenting cells and urothelial cells, resulting in an altered gene expression [9]. This phase corresponds to the early release of so-called inflammatory cytokines (IL-1, IL-6, and IL-8 in humans) which may be responsible for certain adverse effects. Phase 2 consists of recognition of bacterial antigens by CD4^+ ^lymphocytes, which release mainly IL-2 and IFN-γ (TH_1 _response). This cell activation leads to phase 3, consisting of amplification of cytotoxic-populations: CD8^+ ^T cells, gamma-delta lymphocytes, macrophages, and natural killer (NK) cells. All of these cells also release cytokines that may further regulate the BCG response [16].

More recently, studies have shown that mycobacterial DNA contains high amounts of CpG motifs. These CpG motifs induce tumor necrosis factor-related apoptosis-inducing ligand (TRAIL) expression [17] and increase serum levels of mouse keratinocyte-derived chemokine (KC), a functional homolog of human interleukin IL-8 [18]. Urinary TRAIL levels were initially undetectable in BCG therapy patients but levels increased after subsequent treatments. More importantly, patients that responded to BCG therapy had significantly higher urine TRAIL levels, which killed bladder tumor cells *in vitro *versus non-responders. Given this data, it was proposed that TRAIL also plays a role in BCG-induced anti-tumor effects [17]. Nevertheless, BCG's exact mechanism of therapeutic action remains poorly understood. Although systemic reactions have been reported, a more likely scenario is that exposure to BCG results in a local immune response with massive inflammation [5]. This inflammatory infiltrate makes it difficult to analyze the direct effects of BCG on the urinary bladder in BCG treated patients. In order to determine the differences in bladder mucosa and detrusor gene regulation in response to BCG in comparison to those elicited by other inflammatory stimuli (LPS and TNF-α) we sought to differentiate BCG-specific effects from the generalized inflammatory reactivity. For this purpose, we used the C57BL/6 mice because our previous work already defined the time-course of bladder inflammation and urinary cytokine release in response to intravesical instillation of BCG, TNF-α, and LPS [19].

## Results

### Specific Genes

**Figure 1:**
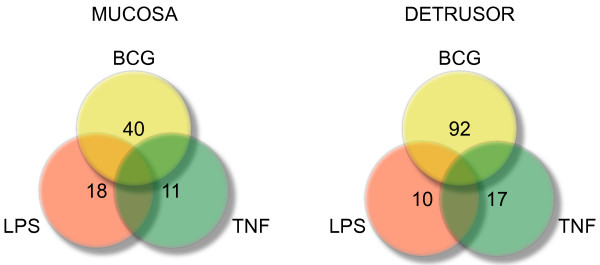
Venn diagram indicating the numbers of Mucosal and Detrusor genes that were classified as BCG-, TNF-, or LPS-specific.

Mucosal and detrusor genes were classified as BCG-, TNF-, or LPS-specific genes and their numbers are represented in the Venn diagram of Figure 1. Genes that were altered by more than one treatment were called unspecific and are not represented (blank spaces in the Venn Diagram). The list of TNF-, LPS-, and BCG-specific genes is presented in Tables S1, S2, and S3, respectively (Additional files 1, 2, and 3 respectively).

### Comparative analysis of networks

**Figure 2:**
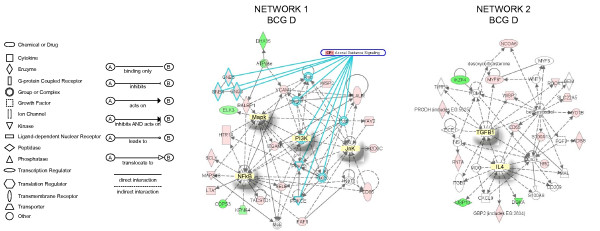
**Networks 1 and 2. BCG-induced specific genes in the detrusor muscle**. Microarray results were normalized by a robust regression analysis and only genes with an expression above a conditional threshold of 0.001 (3 SD above background) were selected for analysis. Next, genes presenting a 3-fold ratio in regard to the control group were entered as focus genes in Ingenuity Pathway Analysis (IPA) for a core analysis which put the datasets in the context of biological processes, pathways, and molecular networks.

**Figure 3:**
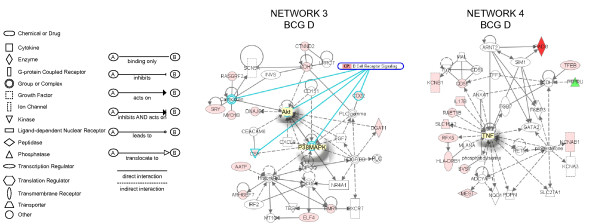
**Networks 3 and 4. BCG-induced specific genes in the detrusor muscle**. Microarray results were normalized by a robust regression analysis and only genes with an expression above a conditional threshold of 0.001 (3 SD above background) were selected for analysis. Next, genes presenting a 3-fold ratio in regard to the control group were entered as focus genes in Ingenuity Pathway Analysis (IPA) for a core analysis which put the datasets in the context of biological processes, pathways, and molecular networks.

**Figure 4:**
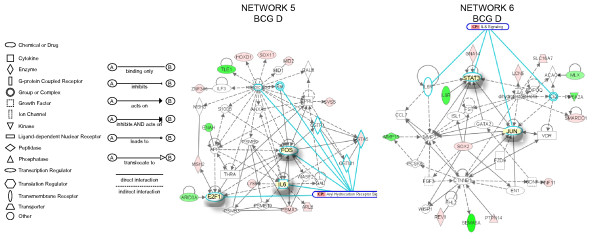
**Networks 5 and 6. BCG-induced specific genes in the detrusor muscle**. Microarray results were normalized by a robust regression analysis and only genes with an expression above a conditional threshold of 0.001 (3 SD above background) were selected for analysis. Next, genes presenting a 3-fold ratio in regard to the control group were entered as focus genes in Ingenuity Pathway Analysis (IPA) for a core analysis which put the datasets in the context of biological processes, pathways, and molecular networks.

Next, the expression values (ratio = Treated/Saline) and the gene bank accession number for each specific genes was entered in the IPA database as focus genes. The interpretation of these datasets in the context of biological processes, pathways, and molecular networks composed the IPA core analysis. Results of the core analysis for BCG-, LPS-, and TNF-α-specific genes in the mucosa and detrusor were entered in a core comparison analysis which allows comparison of changes in biological states across the different treatments and to highlight which biological processes and/or diseases are relevant to each of the treatments. IPA computed a score for each network according to the fit of the set of supplied focus genes (BCG-, TNF-, and LPS-dependent genes). These scores, derived from *p *values, indicated the likelihood that focus genes belonging to a network versus those obtained by chance. A score >2 indicates a ≥ 99% confidence that a focus gene network was not generated by chance alone [20]. Only networks with a score above 20 were selected for further analysis. **Detrusor BCG-specific networks**. From the isolated detrusor muscle, 92 genes were found to be BCG-specific and their networks are represented in Figures 2, 3, 4. **Network 1 **(Figure 2A) received the highest score (50) and assembles 24 BCG-specific genes. This network has in a central position: NF-kappaB, MAP kinase (a NGF/EGF dependent kinase), PI3 kinase, and Jnk and overlays with the axonal guidance canonical pathway (GNB5, GNB4, GNG3, RALBP1, integrin, rac protein, PI3K, PRKD1, PRKCE, PKC, and PDGF). In this pathway, 3 members of the small GTPase family were up-regulated and found upstream of MAP kinase signaling. In contrast, members of the ATPase family such as DEAH (DHX15) were down-regulated. In addition, a gene encoding the mitogen-activated protein kinase kinase kinase 8 was found linking the NF-kappaB, MAP kinase, and ERK kinase (Mek) pathways. A group of genes encoding members of the integrin pathway was also observed and included: PLAUR (plasminogen activator, urokinase receptor), WISP2, VCAM1, and ITGAM (integrin alpha m). This network was correlated with the following functions: cell-to-cell signaling, hematological system development and function, and immune and lymphatic system development and function. **Network 2 **(Figure 2B) presents IL4 and TGFbeta1 in a central position and overlays with a series of canonical pathways (too many to be described). A small GTPase, interferon-inducible, guanylate binding protein 2 (GBP2) was found in this pathway downstream of IL-4 [21]. In addition, several of the BCG-specific genes are related with calcium and β-estradiol metabolism, including: WISP2, ROCK1 (Rho-GTP binding), MYO1B, S100A13, and CD55. This network was correlated with the following functions: cell-to-cell signaling, cellular movement, and cancer. **Network 3 **(Figure 3A) presents Akt and p38MAPK in central positions and overlaps with the B cell receptor canonical pathway (Calmodulin, CSK, P38MAPK, Akt, and CD22). Interestingly, this network presents genes of neuronal origin such as CDH2 (N-cadherin), DNAJ35 (a Hsp40 homolog found in axons), and CTNND2 (catenin or neural plakophilin-related protein). In addition, two genes upstream of Histone H3 were up-regulated by BCG: AATF (an apoptosis antagonist transcription factor) and ARHGEF7 (Rho guanine nucleotide exchange factor). This network was correlated with the following functions: inflammation, dermatological diseases, and cellular growth and differentiation. **Network 4 **(Figure 3B) presents TNF-α in a central position and overlays with a series of canonical pathways (too many to be described). PADI3 was found highly up-regulated (dark red) and encodes peptidyl arginine deiminase, type III, a target of TP53. Genes downstream of TNF-α includes a pro-inflammatory cytokine IL-17, RAET1B (retinoic acid early transcript beta), HLA-DRB1 (a major histocompatibility complex, class II), RFX5 (an MHC regulatory factor), and CD55 (a member of the complement system). In this network, TFEB was up-regulated and this gene encodes a transcriptional factor expressed in the urothelium and detrusor muscle that responds to bladder inflammation [22]. This network was correlated with the following functions: cancer, cell-to-cell signaling, and immunological disease. **Network 5 **(Figure 4A) presents FOS, IL-6, and the transcription factor E2F1 in a central position and overlays with the aryl hydrocarbon receptor canonic pathway (GSTA5, GSTP1, GSTM1, Rar [all trans retinoic acid receptor], FOS, IL-6, E2F1, and retinoic acid). Interestingly, a group of genes involved in retinoic acid metabolism was highlighted, and included: ILF3 (interleukin enhancer binding factor 3), HOXD1 (homeobox D1), SOX, MID2, and GSTA5 (glutathione S-transferase A5). This network was correlated with the following functions: cellular signaling, cancer, and cellular growth and proliferation. **Network 6 **presents STAT3 and JUN in a central position and overlaps with the IL-6 canonic pathway (IL6R; JUN; Ck2; STAT3), (Figure 4B). A small GTPase (GNA14) was found upstream of STAT3. Interestingly, a group of genes involved in the dihydrotestosterone metabolism was highlighted: LCN5, SLC16A7, and SMARCC1. In addition, genes involved in the regulation of MMP2 (gelatinase A), such as MMP15 were found down-regulated by BCG treatment. Downstream of CTNNB1 (catenin), SEMA5A, an axon guidance molecule, was down-regulated by BCG. This network was correlated with the following functions: cellular development, development disorder, and cellular growth and proliferation.

### Mucosal BCG-specific networks

**Figure 5:**
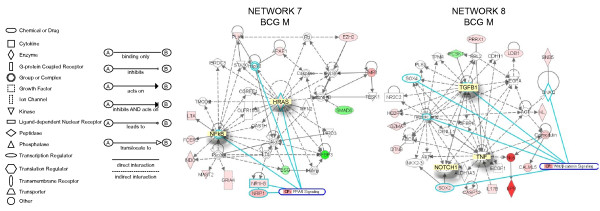
**Networks 7 and 8. BCG-induced specific genes in the bladder mucosa**. Microarray results were normalized by a robust regression analysis and only genes with an expression above a conditional threshold of 0.001 (3 SD above background) were selected for analysis. Next, genes presenting a 3-fold ratio in regard to the control group were entered as focus genes in Ingenuity Pathway Analysis (IPA) for a core analysis which put the datasets in the context of biological processes, pathways, and molecular networks.

**Figure 6:**
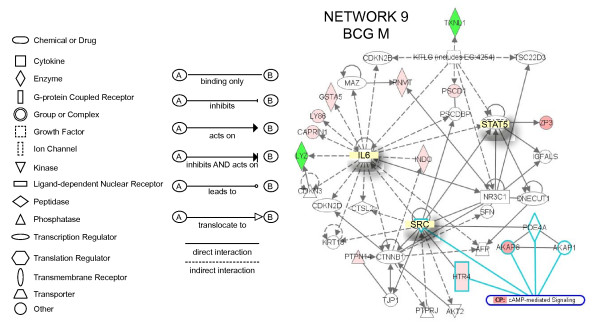
**Networks 9. BCG-induced specific genes in the bladder mucosa**. Microarray results were normalized by a robust regression analysis and only genes with an expression above a conditional threshold of 0.001 (3 SD above background) were selected for analysis. Next, genes presenting a 3-fold ratio in regard to the control group were entered as focus genes in Ingenuity Pathway Analysis (IPA) for a core analysis which put the datasets in the context of biological processes, pathways, and molecular networks.

**Network 7 **(Figure 5A) presents NF-kappaB and HRAS in central positions and overlays with the PPAR canonic pathway (NF-kappaB, NRIP1, NR1H3, HRAS, Hsp90). Genes correlated with NF-kappaB include INDO (a indoleamine-pyrrole 2,3 dioxygenase that represents a target for IFN-γ), which is responsible for regulating tryptophan metabolism, FCER2 (Fc fragment of IgE receptor), MAST2 (member of IL-4 signalling, a microtubule associated serine/threonine kinase), GRIA4 (glutamate receptor), and LTA (member of TNF super family). In this pathway, IGFBP3, SMAD6, and BSG were found to be down-regulated. This pathway was correlated with the following functions: gene expression, cancer, and cell cycle. **Network 8 **(Figure 5B) has TGFB1, NOTCH1, and TNF-α in a central position, and overlaps with the Wnt/β-catenin canonic pathway (GNAQ; TGFB1, NOTCH1, SOX4, and retinoic acid). Genes highly up-regulated (dark red) included: NOS (neuronal endothelium-derived relaxation factor-forming enzyme), and SPR (an aldo-keto reductase that is part of the arginine metabolism). Additional genes of the TNF-α pathway included the pro-inflammatory cytokine IL-17 and caspase 1. Another small GTPase (GNB5) was part of this pathway. In addition, several genes involved in retinoic acid metabolism were highlighted, including: GZMA (granzyme A), and HEPBP1 (a heme binding protein). This pathway was correlated with the following functions: cellular development, connective tissue development and function, skeletal muscular system development and function. **Network 9 **(Figure 6) has IL-6, STAT5, and SRC in a central position. This pathway overlaps with the cAMP-canonical pathway (HTR4, SRC; PDE4A; AKAP8; and AKAP1) and G-Protein canonical pathway (SRC; AKT2; HTR4; and PDE4A). Highly up-regulated genes (dark red) included: AKAP8 (a kinase anchor protein 8) and ZP3 (zona pellucida glycoprotein 3). Down-regulated genes included LYZ (a lysozyme) and TXNL1 (thioredoxin-like 1, a regulator of insulin). This pathway was correlated with the following functions: cellular growth and proliferation, cancer, and cell-to-cell signaling.

### TNF-α-specific genes

**Figure 7:**
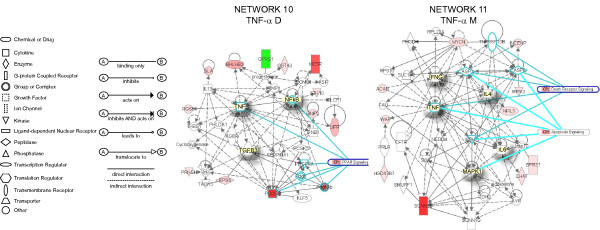
**Networks 10 and 11. TNF-α-induced specific genes in the detrusor muscle and bladder mucosa**. Microarray results were normalized by a robust regression analysis and only genes with an expression above a conditional threshold of 0.001 (3 SD above background) were selected for analysis. Next, genes presenting a 3-fold ratio in regard to the control group were entered as focus genes in Ingenuity Pathway Analysis (IPA) for a core analysis which put the datasets in the context of biological processes, pathways, and molecular networks.

**Network 10 **presents detrusor genes specifically up-regulated by TNF-α (Figure 7A). This network presents TNF-α, NF-kappaB, and TGFB3 in central positions and overlays with PPAR signaling canonical pathway (TNF-α; PDGF; PDGFR, PDGF Ab; PDGFRA; and NF-kappaB). Genes highly up-regulated (dark red) included: PDGFB, and MC5R (melanocortin 5 receptor). OPRS1 (opioid receptor, sigma) was down-regulated by TNF-α. This pathway was correlated with the following functions: cancer, post-translational modification, and cell death. **Network 11 **presents mucosal genes specifically up-regulated by TNF-α (Figure 7B). This network has TNF-α, IFNγ, IL-4, IL-6, and MAPK1 in central positions and overlays with the death receptor canonical pathway (IFNγ, TNF-α, IKBKB, CASP3, CASP8, and TNFRSF1DB) and the apoptosis signaling canonical pathway (TNF-α, IKBKB, CASP3, CASP8, and MAPK1). The gene encoding a sodium channel (SCNN1B) was found highly up-regulated (dark red). This pathway was correlated with the following functions: cancer, cell death, and reproductive system disease.

### LPS-specific genes

**Figure 8:**
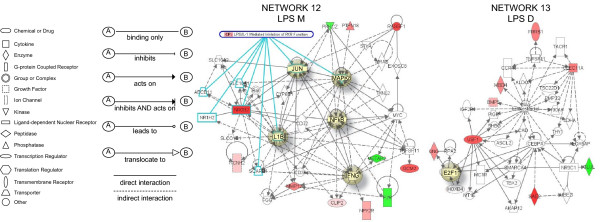
**Networks 12 and 13. LPS-induced specific genes in the bladder mucosa and detrusor muscle**. Microarray results were normalized by a robust regression analysis and only genes with an expression above a conditional threshold of 0.001 (3 SD above background) were selected for analysis. Next, genes presenting a 3-fold ratio in regard to the control group were entered as focus genes in Ingenuity Pathway Analysis (IPA) for a core analysis which put the datasets in the context of biological processes, pathways, and molecular networks.

**Network 12 **(Figure 8A) presents mucosal genes specifically up-regulated by LPS and has NF-kappaB, MAPK8, JUN, IL-1β, and IFNγ in central positions. This network overlaps with the canonical pathway of LPS/IL-1-mediated inhibition of RXR receptors (JUN, MAPK8, SLC10A1 [solute carrier family 10], NR0B2 [nuclear receptor subfamily 0, group B, member 2], ABCB11 [ATP-binding cassette], IL-1B, SCARB1 [scavenger receptor class B, member 1], and NR1H2 [nuclear receptor subfamily 1, group H, member 2]). Genes highly up-regulated included: NR0B2, MMP12, PTPN18 (protein tyrosine phosphatase, non-receptor type 18), and RASSF1 (Ras association [RalGDS/AF-6] domain family 1). This pathway was correlated with the following functions: cell signaling, genetic disorder, and hepatic system disease. **Network 13 **(Figure 8B) presents detrusor genes specifically up-regulated by LPS and overlays with a series of canonical pathways (too many to be described). The transcription factor E2F1 is in a central position. This pathway presents several highly up-regulated genes (dark red), including the transcription factors USF1 and BMP5. In addition, a series of β-estradiol related genes were present in this network. This pathway was correlated with the following functions: cellular growth and proliferation, cancer, and cellular development.

### Target validation of BCG-dependent genes by Q-PCR of Chromatin Immunoprecipitation (CHIP)-Based Assays

**Figure 9:**
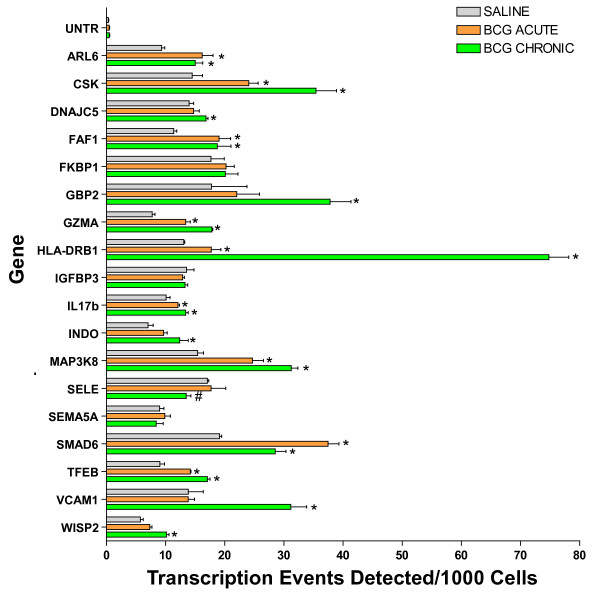
**Target validation of selected genes and small GTPases by ChIP/Q-PCR.** Female C57BL/6J mice (n = 20 per group) were instilled with 200 μl of one of the following substances: BCG (total dose of 1.35 mg) or pyrogen-free saline on days 1, 7, 14, and 21, as described above. Mice were euthanized 24 hours after a single instillation (**BCG acute**) or 7 days after 4 weekly instillations (**Control **and **BCG chronic**). Bladders were exposed briefly to formaldehyde for cross-linking of the proteins and DNA together, followed by sonication to fragment the DNA. An antibody against RNA polymerase II (Abcam) was then used to precipitate the DNA transcriptome. The final ChIP DNAs were then used as templates for Q-PCR reactions using primer pairs specific for each gene of interest (Table S4; (Additional File 4). Q-PCRs were run in triplicate and the averaged Ct values were transferred into copy numbers of DNA using a standard curve of genomic DNA with known copy numbers. The resulting transcription values for each gene were also normalized for primer pair amplification efficiency using the Q-PCR values obtained with input DNA (un-precipitated genomic DNA). Results are presented as "transcription events detected per 1000 cells" for each gene tested. Error bars correspond to standard deviations from the triplicate Q-PCR reactions. Control represents an un-transcribed region of the genome. Asterisks indicate a statistically significant increase (p < 0.05) between BCG-treated and control and a pound sign indicates a statistically significant decrease (p < 0.05) between BCG-treated and control.

The validity of the predictions of transcriptome analysis was tested using ChIP-Q-PCR because this is more definitive than confirmation of RNA levels and shows genes that are being actively transcribed. We selected genes that appeared to show a clear change from the transcriptome to be tested by ChIP-Q-PCR. For this purpose, additional mice were treated with saline or acutely and chronically with BCG, the urinary bladder was removed and an antibody against RNA polymerase II (Abcam) was then used to precipitate the DNA transcriptome. Q-PCR was performed with primer pairs listed in Table S4 (Additional File 4). Figure 9 depicts the averaged transcription events detected per 1000 cells for each gene tested and their standard deviations. With the exception of SEMA5A, FVBP1, IGFBP3, and SELE, these results indicate that chronic treatment with BCG and to a lesser extent acute BCG induced up-regulation of the following genes as was predicted by the mRNA-level analysis: ARL6, CSK, DNAJC5, FAF1, GBP2, GZMA, HLA-DRB1, IL17b, INDO, MAP3K8, SMAD6, TFEB, VCAM1, and WISP2.

### Participation of NF-kappaB in the bladder responses to BCG

**Figure 10:**
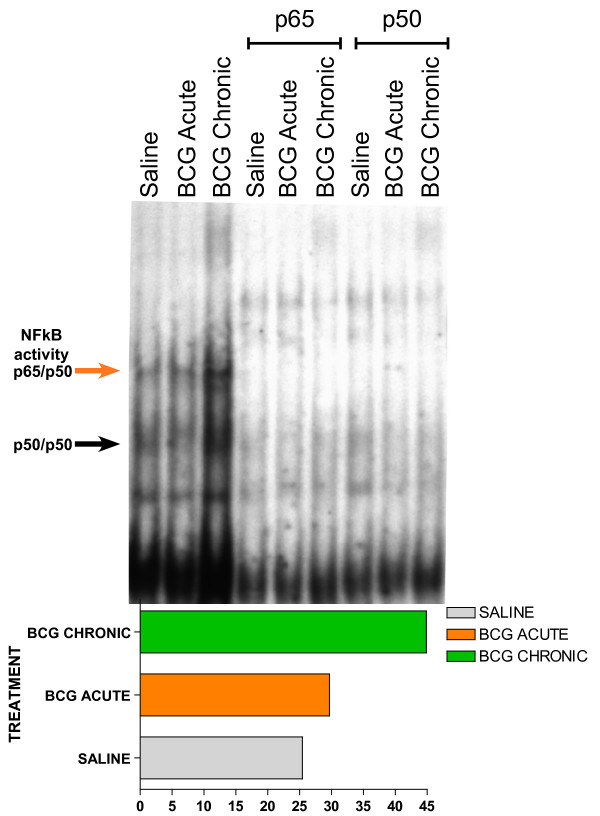
**NF-kappaB electrophoretic mobility shift assays (EMSA)**. Anesthetized C57BL6 female mice were instilled with 200 μl of one of the following substances: BCG (TheraCys^®^-Aventis-Pasteur; total dose of 1.35 mg) or pyrogen-free saline on days 1, 7, 14, and 21, as described above. Mice were euthanized with pentobarbital (200 mg/kg, i.p.) 24 hours after a single instillation (**BCG acute**) or 7 days after 4 weekly instillations (**BCG chronic**). Bladder mucosa nuclear extracts were incubated with 2 ng of [^32^P] NF-kappaB double-stranded probe. Supershift reactions were pre-incubated for 20 minutes with antibody against p65 or p50. Insert graph represents the quantification of p50/p65 NF-kappaB activity (indicated by red arrow) using ImageJ Software. Statistical differences were determined using GraphPad Software.

**Figure 11:**
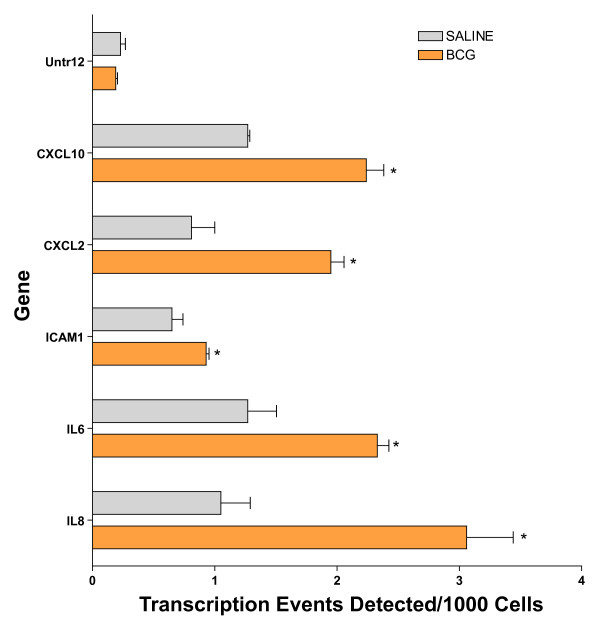
**BCG-induced increased NF-kappaB activity in J82 human cancer cell line**. Human urothelial cancer cell line (J82) was exposed to BCG or saline for 24 hours and the cells were frozen and prepared for ChiP-Q-PCR assays. In this experiment the chromatin was precipitated with a p65 antibody. The final ChIP DNAs were then used as templates for Q-PCR reactions using primer pairs specific for each genomic region of interest. Q-PCR was carried out using Taq polymerase (iQ SYBR Green Supermix, Bio-Rad). Details of the primer sequences and the Genebank accession numbers are given in Table S4 (Additional File 4). Results are presented as "transcription events detected per 1000 cells" for each gene tested. Error bars correspond to standard deviations from the triplicate Q-PCR reactions. Control represents an un-transcribed region of the genome. Asterisks indicate a statistical significant difference (p < 0.05) between saline- and BCG-treated cells.

Because NF-kappaB played a central role in several gene networks here described, we predicted that BCG induces NF-kappaB translocation by using EMSA. The results presented in Figure 10 indicate that chronic BCG indeed increases the binding of NF-kappaB. To further confirm this finding, we tested whether the BCG-induced increased binding of NF-kappaB would lead to up-regulation of transcripts known to possess a p65 binding site. For this purpose, the human urothelial cancer cell line (J82) was exposed to BCG or saline for 24 hours and the cells were frozen and prepared for ChiP-Q-PCR assays. In this experiment the chromatin was precipitated with a p65 antibody and the following genes were found to be up-regulated by BCG: CXCL10, CXCL2, ICAM1, IL-6, and IL-8 (Figure 11). 

### Morphology and immunohistochemistry of urinary bladders instilled with BCG-GFP

**Figure 12:**
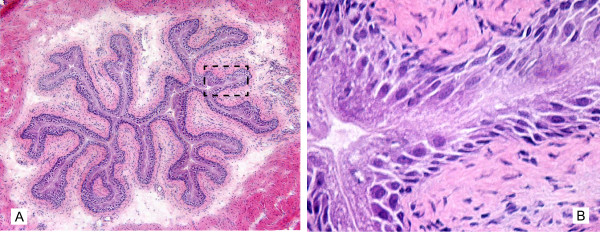
**Morphology of the mouse urinary bladder secondary to rBCG-GFP instillation**. 1.5 × 10^7 ^CFU of GFP-expressing rBCG in 200 microliters were instilled into the bladder of anesthetized mice, as described in material and methods. Twenty-four hours after instillation, the bladder were removed and frozen for morphological analyzes. H&E sections were photographed at 200× (Figure 12A) and 400× (Figure 12B). For composite H&E pictures (Fugure 12 A), at total of 9 photos were taken at 200× and automatically merged in Adobe Photoshop^® ^CS3 extended [89]. Note that 200 microliters instilled into the bladder of anesthetized mice does not disrupt the urothelium integrity.

Because of the large volume (200 μl) of BCG that was administered in mice relative as compared to humans, we further investigated whether acute instillation of BCG-GFP at bladder capacity does not result in urothelial injury and microscopic extravasation of BCG or would cause artifacts, including forcing bacteria into the urothelium. For this purpose, mice were euthanized 24 hours following bladder instillation with BCG-GFP, the bladders were removed and frozen, and were stained with H&E or processed for fluorescence immunohistochemistry. Figure 12A is a representative photomicrograph covering the whole urothelial surface. This is a composite of nine pictures at original magnification of 200× that was electronically merged. This picture shows that in our conditions the instillation of BCG-GFP does not cause any urothelial injury. Figure 12B is a high magnification (400×) of the dotted black box highlighted in Figure 12A and confirms the integrity of the urothelial cell layer.

**Figure 13:**
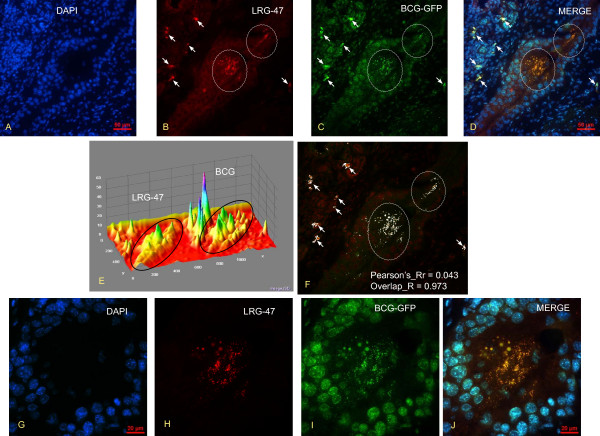
**Co-localization of BCG-GFP with LRG47-positive vesicles in the mouse bladder urothelium**. Figures 13 A-D are representative photomicrographs obtained 24 hours after bladder instillation of BCG-GFP (1.5 × 10^7^ CFU) into the bladder of anesthetized mice (see material and methods) and Figures 13 G-J are high magnifications of the same area depicted in Figures A-D.  Figures 13 A and 13 G are DAPI [4',6-diamidino-2-phenylindole] that was used to highlight the nucleus. Figures 13B and 13H were stained with LRG-47. Figures 13C and 13I indicate the presence of BCG in the urothelium as indicated by the fluorescence of GFP in two major areas delimited by white circles. Figures 13D and 13J are merged photograph indicating that BCG-GFP is co-localized in vesicles positives for LRG-47 (yellow). White arrows in Figures 13 A-D point to GFP and rhodamine signals that are co-localized in the merge picture (Figure 13D). Next, pictures 13B and 13C were submitted to image analysis by the Interactive 3D Surface Plot [90], a plug-in for ImageJ software [23]. Figure 13E represents the Integrative 3D surface that translates the luminance of the images in Figure 13 B-C as height for the plot using the nearest neighbor sampling. For co-localization of BCG-GFP and LRG47-Rhodamine, pictures were converted to 8-bit gray scale images (fluorescence intensity range: 0 < 255) using Image J software [23] and the analysis was performed with the co-localization-finder plug-in [24]. The results presented in Figure 13F were electronically generated by the software resulting in a merged picture with white areas indicating the co-localization between BCG and LRG-47-positive vesicles and the Pearson's_correlation and overlap coefficient are provided. These results indicate that instillation of BCG-GFP into the mouse bladder, in the conditions described in this manuscript, does not result in urothelial injury and microscopic extravasation of BCG or cause artifacts, including forcing bacteria into the urothelium.

Next, we used fluorescence immunohistochemistry to demonstrate that, in our laboratory conditions, instillation of BCG-GFP does not cause any microscopic extravasation of BCG or cause artifacts, including forcing bacteria into the urothelium. Figures 13A–D are representative photomicrographs obtained 24 hours after bladder instillation of BCG-GFP. Figure 13A is a DAPI [4',6-diamidino-2-phenylindole] that was used to highlight the nucleus. Figure 13B was stained with LRG-47. Figure 13C indicates the presence of BCG in the urothelium as indicated by the fluorescence of GFP. It has to be noted that not only fluorescence if enough for recognition of GFP. The size is also fundamental since the urinary bladder presents high background fluorescence. In this context, specific GFP indicative of BCG are highlighted in two areas of the urothelium contained within white circles (Figure 13C). Small particles positively labeled with GFP can be noticed in those areas. In the merged picture, presented in Figure 13D, it can be appreciated that most of background fluorescence coincides with DAPI staining (blue) whereas the BCG-GFP is co-localized in vesicles positives for LRG-47 (yellow). White arrows in figures 13A–D point to signals that are co-localizes in the merge picture (Figure 13D). This set of figures illustrates also the absence of GFP in any portion of the urinary bladder outside LRG-47 vesicles. Next, pictures 13B and 13C were submitted to image analysis. Figure 13E represents the Integrative 3D surface which translates the luminance of the image in Figure 13B–C as height for the plot using the nearest neighbor sampling, next the viewing position of the plot was adjusted for the nest visualization of the surface plot. The black circles indicate the central areas of Figure 13B–C with the highest density of BCG and LRF-47. Next, the co-localization of BCG-GFP and LRG47-Rhodamine was performed by converting the Figures 13B and 13C to 8-bit gray scale images (fluorescence intensity range: 0 < 255) using Image J software [23], assigned as red and green channels, and the analysis was performed with the co-localization-finder plug-in [24]. The results presented in Figure 13F were electronically generated by the software resulting in a merged picture with white areas indicating the co-localization between BCG and LRG-47-positive vesicles. The results were also automatically tabled and provided the following parameters: Pearson's_correlation Rr = 0.0431; overlap coefficient R = 0.973628; contribution of red channel k1 = 2.986; contribution of green channel k2 = 0.371; slope = 0.0854; intercept = 99.72. Next, the central area of Figures 13A–D was photographed at high magnification. The results presented in Figures 13G–J illustrate the findings of the image analysis indicating an almost complete overlap between BCG and LRG-47 (yellow dots and vesicles in Figure 13J illustrate the image analysis results indicating an overlap R = 0.0973) and that no BCG was found outside LRG-47-positive vesicles. Since LRG-47 vesicles are part of the endocytic pathway, it is very unlikely that the bacterium was mechanically forced into these vesicles. Together these results indicate that instillation of BCG-GFP into the mouse bladder, in the conditions described in this manuscript, does not result in urothelial injury and microscopic extravasation of BCG or cause artifacts, including forcing bacteria into the urothelium.

## Discussion

The present study was set forth to determine unique genes modulated by BCG in the bladder target organ apart from a general inflammatory response. In this context, treatment-specific genes were used to query the IPA in order to construct molecular networks. Our results indicate that some unique functions are involved in bladder responses to BCG. This includes a whole new hypothesis that BCG interferes with several genes participating in axonal guidance signaling (network 1). The latter includes a family of small GTPases that along with integrin, protein kinase D1, PKC, and platelet-derived growth factor regulate axonal guidance. Although this finding potentially linking BCG to axonal guidance is unique, activation of small GTPases genes by intravesical instillation of BCG into the mouse bladder was just demonstrated by our laboratory [25] and we extended the results into the present work, indicating that acute BCG does co-localize with LRG47 vesicles. However, it remains to be determined whether the same genes exert axonal guidance properties in the urinary bladder.

Another unique and exciting finding was that the primary function of Network 1 was related to the development of the immune and lymphatic systems, including formation of lymphoid tissue and Peyer's patches (LTA [26,27] and VCAM1 [28,29]), size of lymphoid organs (CD86, PRKCE, and VCAM1 [28]), lymph node development [LTA [27]], degranulation of the lymphatic system (PLAUR [30], and CD86), and dilation of lymph vessels (ELK3) [31]. Our results suggest that BCG induces a series of genes that may lead to bladder lymphangiogenesis since their role in the development and function of the lymphatic system has been established. These included VCAM1 (vascular cell adhesion molecule 1), a cell surface glycoprotein implicated in the development of lymphoid tissues [28] and in a variety of chronic inflammatory conditions, making its expression and function a target for therapeutic intervention [32]. Interestingly, a role for NF-kappaB regulating VCAM1 expression has been proposed [33]. Another gene encodes the LTA (lymphotoxin alpha, TNF-α superfamily, member 1) which is involved in Peyer's patch development [26] and is essential for inflammation-induced lymphangiogenesis. Indeed, genetic deletion of lymphotoxin beta receptor or lymphotoxin alpha abrogated development of lymphatic vessels in the inflamed areas in the thyroid but did not affect development of neighboring lymphatics [34]. Also LTA seems to be important in the local lymphoid tissues and lymph node development [27]. Unlike other cytokines, lymphotoxin alpha/beta regulates the development of intestinal lymphoid organs, including Peyer's patches (PPs) and mesenteric lymph nodes (MLNs). In addition, intestinal inflammation is suppressed by inhibition of LTB signaling [35]. A role for LTB signaling has been proposed in initiating immune responses both dependent and independent of its role in maintaining the organization of lymphoid tissues [36]. Coincidently, lymphotoxin also activates the NF-kappaB pathway [37]. To the best of our knowledge, there is only a single manuscript indicating that another bacteria, *Mycoplasma pulmonis *induces lymphangiogenesis [38]. Our results suggest that BCG may have a similar role. It remains to be determined if alteration of the lymphatic system is part of the beneficial responses of the bladder to BCG treatment. Another finding of the present work was the participation of BCG-specific genes in networks involved in the axonal guidance canonical pathway (Network 1). In addition, Network 3 highlights genes of neuronal origin, including: CDH2 (N-cadherin), DNAJ35 (a Hsp40 homolog found in axons), and CTNND2 (catenin or neural plakophilin-related protein). The combination of these two findings raises the hypothesis that BCG treatment may impact the nervous system, possibility explaining its therapeutic role in interstitial cystitis. There are some reports already in the literature supporting this hypothesis: the immune responses to BCG mediates axon damage in rodents [39]; bolus injection of BCG in rats alters the brain vasculature and predisposes subjects to thrombosis and hemorrhage [40], and the supernatant of BCG-stimulated murine peritoneal macrophages alters neurite development in organotypic culture [41]. Not surprising, several networks (2, 3, 5, 7, and 9) overlap with pathways involved in cancer. However, each one of the cancer networks provides a specific association of genes and transcription factors that form hypothesis that can be tested to further determine the role they might play in the anti-cancer action of BCG. Network 2 has TGFB1 and IL4 driving several genes known to be involved in cancer development but also known to negatively regulate potentially tissue destructive immune responses. In contrast, Network 3, which is involved in B cell receptor signaling, has Akt and P38 MAPk in a central position. Network 5 has retinoic acid in a central position. Network 7 has HRAS and NF-kappaB in a central position and overlaps with the PPAR signaling. Finally, Network 9 has IL6 in a central position and genes such as INDO that is a known target of IFN-γ. Interestingly, both networks associated with TNF-α-specific genes (Networks 10 and 11) overlap and have as top functions cancer and cell death. Regarding the networks associated with LPS-specific genes (Networks 12 and 13); at least the one found in the bladder mucosa was associated with the canonical pathway of LPS/IL1 mediated alterations of nuclear RXR receptors (including retinoic acid and PPARs). The identification of these various networks bespeaks the complexity of BCG-induced reactions.

Regarding putative transcription factors (TF), TFEB (Network 4) is known to be expressed in the mouse bladder urothelium and its activity is increased during bladder inflammation [39]. In addition, our ChIP-Q-PCR results confirmed that BCG induces up-regulation of the gene encoding this TF. Another TF is E2F1 and networks 5 and 13 present this transcription factor in a central position. Interestingly, an E2F1-dependent increase in transcription was found in U1 bladder cancer cells emerging from quiescence upon serum stimulation [42]. E2F1 promoter activity in quiescent cells was associated with recruitment of NF-kappaB. NF-kappaB was replaced largely by E2F1 in concert with gene activation during the early stage (12 h) of serum stimulation. The finding that NF-kappaB is expressed in a central position in several inflammatory networks (1, 7, 10, and 12), corroborates with the proposed role of NF-kappaB as a link between chronic inflammation and cancer [43]. NF-kappaB seems to play a major role in gene regulation in urothelial cells and several recent studies reveal a role for NF-kappaB on the responses of these cells to BCG [12,44,45]. Indeed, BCG induced IL-6 expression by human transitional cell neoplasms requires NF-kappaB and AP-1 [46,47]. Others have presented evidence that BCG activates nuclear translocation of the transcription factor NF-kappaB, since pretreatment of with sulfasalazine, an inhibitor of NF-kappaB activity, blocked the ability of BCG to induce IL-8 secretion [48,49]. Others have shown that BCG induces CXCL8 production in epithelial cells by a mechanism involving NF-kappaB [50]. Finally, several laboratories have provided additional evidence of NF-kappaB involvement in urinary bladder cancer [51] and the benefits of drugs derived from curcumin, an inhibitor of NF-kappaB [52]. Recently, a functional insertion/deletion polymorphism has been identified in the promoter region of NFKB1 that is being proposed as a useful marker for the identification of patients with high risk of recurrence of superficial bladder cancer [53]. Our present findings using EMSA and ChIP-Q-PCR confirmed that both the mouse urinary and the J82 urothelial cancer cell line respond to BCG treatment with an increased translocation of NF-kappaB.

The results here presented have to be taken in light of the choices made to provide a manageable focus for this paper. This study was carried out at a single time point. We understand that this time point may not identify preceding or proceeding events as we indicated previously [54-56]. Therefore, the results of regulatory network here presented should be viewed as a snapshot of the bladder transcriptome. Nevertheless, genes identified as important at this time point can now be followed over time by other techniques. The study also focused on the responses that are unique to BCG, which loses sight of the larger picture of the response common to all three stimuli of inflammation. The common pathways have been considered previously [56,57]. Another short coming of this type of analysis was to exclude initially genes that are commonly up-regulated by the 3 different stimuli. The rationale for the latter was to focus the study on genes specifically regulated by BCG in contrast to the other stimuli. By doing this, we did not discuss or further analyze the common genes involved in bladder responses to inflammation. Although genes responding to more than one treatment were not considered, when treatment-specific genes were assembled in molecular networks, the network themselves shared some overlap of functions.

One of the family of genes induced specifically by BCG in the bladder mucosa belong to small GTPases (Network 1) and GBP2 on Network 2. These results confirmed our recent reports using suppressive subtraction hybridization that the small GTPases are upregulated in the bladder mucosa in response to BCG [25]. Here we went further to verify whether BCG-GFP co-localizes with one of the small GTPases. Indeed, our results indicate that BCG-GFP has almost 100% coincidence with LRG-47-positive vesicles (Figure 13). The association of p47 GTPases with membranous compartments such as the endoplasmic reticulum and the Golgi, implicate these molecules in intracellular membrane trafficking or processing [58] and that mechanical stimuli alone could not be responsible for our findings. In addition, products of these genes are known to coordinate cellular defense against intracellular pathogens such as *Mycobacteria *[59,60], possibly by promoting acidification of the phagosome [61] and elimination of the intracellular pathogen [62]. p47 GTPases are one of the most effective cell-autonomous resistance systems known against intracellular pathogens in the mouse [60,61], and have been shown to be essential for immune control *in vivo *of *Listeria monocytogenes*, *Toxoplasma gondii*, and *Mycobacterium tuberculosis *(*Mtb*) [63]. In addition, mice lacking LRG-47 failed to control *Mtb *replication [63]. Thus, LRG-47 may serve as a critical vacuolar trafficking component used to dispose of intracellular pathogens like *Mtb *[63]. Therefore, it is expected that this group of GTPases may limit the activity and survival of BCG in the mouse bladder. In addition to limiting BCG activity, this group of GTPases is part of the inflammatory response. In particular, human GBP-1 and GBP-2 modulate the endothelial cell responses to inflammatory cytokines [64,65]. Taken together, these results suggest that in the case of BCG vaccine, these GTPases may act as a controlled-release mechanism for the agent infecting the bladder.

The question arises as to the relevance of these findings to the clinical use of BCG and particularly whether the response of normal urothelium to BCG is relevant to cancer treatment. First, the major difference is that the mice used in the present work do not have cancer, and, therefore, our results are most relevant to the response of the bladder to BCG and provide no information on cancer-specific responses. A second potential difference is whether the larger volume of BCG administered relative to bladder volume in these mice as compared to human clinical use might cause artifacts, including forcing bacteria into the urothelium. Our previous studies using pressure monitoring showed that at 0.2 ml, the pressure was less than 10% of that required to produce overdistention.

The literature is remarkably mute on the presence of BCG within the urothelium within 24 hours of administration, and studies that reported on the absence of BCG in the bladder were performed a week after BCG administration, well after the responses documented here occurred and after much of the urothelium had sloughed [66,67]. We suggest that a deeper understanding of the sequence of the response of humans with bladder cancer treated with BCG may require biopsies within 24–48 hours to determine whether BCG enters the urothelium and then produces responses similar to those that occur in the mouse urothelium reported here. However, it is not practical to biopsy a human being, 24 hours after administering BCG. In this context, the animal model here described, with all its limitations, can facilitate investigations of the basic processes and mechanisms involved in urinary bladder responses to intravesical BCG instillation.

## Conclusion

In conclusion, the present research approach identified genes involved in the responses to intravesical BCG therapy in the bladder target organ apart from those involved in the responses to LPS and TNF-α and suggested novel mechanisms involved in the responses to BCG. The major findings of the present work suggest that BCG is involved in the development and function of immune system and lymphatic vessels. Interestingly, the present work confirms our recent findings [25] that BCG also up-regulates genes encoding several small GTPases, such as LRG47 [25] that are responsible for an organism's defense to intracellular pathogens. It remains to be determined whether over-expression of small GTPases would limit the survival of BCG within the urinary bladder or whether it will contribute to a controlled-release mechanism for the agent infecting the bladder.

## Methods

### Animals

All animal experimentation described here was performed in conformity with the "Guiding Principles for Research Involving Animals and Human Beings" (OUHSC Animal Care & Use Committee protocol #05-081I and 05-088). Ten to twelve week-old C57BL/6 female mice (The Jackson Laboratory; Bar Harbor, ME) were anesthetized, transurethrally catheterized as previously described, and instilled with 200 μl at a pressure not exceeding 5 cmH20. Using this procedure in previous experiments, we found no evidence of disruption of urothelial layers due to over distention [19,22,25,57,68-70]. Mice were instilled with one of the following substances: BCG (TheraCys^®^-Aventis-Pasteur; total dose of 1.35 mg [71]), *E. coli *LPS (strain 055:B5; 100 μg/ml [55,56,72], TNF-α (1 μg/ml) [19], or pyrogen-free saline on days 1, 7, 14, and 21. Seven days after the last instillation, mice were euthanized with pentobarbital (200 mg/kg, i.p.), and the bladders removed, placed in RNA *later™ *(Ambion) and visualized under a dissecting microscope (Nikon SMZ 1500). As previously described [57], the urothelium along with the submucosa were separated from the detrusor muscle and used in subsequent experiments.

### Minimum Information About Microarray Experiments – MIAME [73]

#### Objective

To determine the time course of gene-expression in urinary bladders in response to chronic intravesical instillation of saline, BCG, TNF-α, or LPS.

### Array design

Mouse plastic 5 K Arrays (Clontech, Palo Alto, CA, Cat. #634809). For a complete list of genes present in this array see reference [74].

### Animal Numbers

This experiment involved: twelve mice per group; three different biological replicates; four treatments: saline, BCG, TNF-α, and LPS; and two tissue layers: urothelium/submucosa and detrusor muscle; for a total of 288 mice.

### Sample preparation for cDNA Expression Arrays

We used the same technology as we described before [54,56,75-77]. Briefly, the bladder mucosa (n = 12) and the detrusor muscle (n = 12) of the bladders from each group were homogenized together in Ultraspec RNA solution (Biotecx Laboratories Inc. Houston, TX) for isolation and purification of total RNA. Three biological replicates were used per group. RNA was DNase-treated according to manufacturer's instructions (Clontech Laboratories, Palo Alto, CA), and the quality of 10 μg was evaluated by denaturing formaldehyde/agarose gel electrophoresis. RNA yielded a ratio 260/280 > 1.8, relatively intact ribosome RNA, and minimum low molecular weight degradation.

### Mouse cDNA Expression Arrays

cDNA probes were prepared from DNase-treated RNAs obtained from each of the experimental groups. Five μg of DNase-treated RNA was reverse-transcribed to cDNA and labeled with [α-^33^P]dATP, according to the manufacturer's protocol (Clontech, Palo Alto, CA). The radioactively labeled complex cDNA probes were hybridized overnight to Atlas™ mouse plastic 5 K arrays (Clontech, Palo Alto, CA) using ExpressHyb™ hybridization solution with continuous agitation at 68°C. After two high-stringency washes, the hybridized membranes were exposed (at room temperature) to a ST Cyclone phosphor screen overnight. Spots on the arrays were quantified by BD AtlasImage™ 2.7 software (Clontech, Palo Alto, CA).

#### Array normalization

Normalization among array experiments was based on the fact that spot intensities from genes not expressed by biological specimens constitute noise and are therefore randomly distributed. The method models the signals from non-expressed genes to a normal distribution using an iterative nonlinear curve fitting procedure. A two-step normalization procedure was performed, as described [77]. The first step was the determination of the parameters of the background of the array – average (Av) and standard deviation (SD) of normally distributed low level expressions in the array. A normalized score, "S," was obtained [S = (PV - Av)/SD], where PV is the original pixel value for the spot, and Av and SD are the mean and standard deviation of the set of background spots. In the normalized array, the distribution of **S **has mean of zero and **SD **= 1 over the set of background genes [77]. We accepted all genes expressing 3 SDs above the mean background that were used for "adjustment" as described below.

#### Adjustment of the normalized profiles to each other by a robust regression analysis

In this analysis potential outliers were identified and their contribution to the calculations down-weighted in an iterative manner. All expression profiles of both control and treated groups were then re-scaled to the averaged control group. Outliers were thereafter, determined as having deviations not associated with the normal distribution. Robust regression analyses was used to exclude the influences of potential outliers [77]. Only genes that were expressed 3 SDs above the average were submitted to further analysis and the ratio of gene-expression between LPS-, TNF-, BCG- and saline-challenge was obtained. Genes expressing a ratio greater than 3.0 were selected and classified as BCG-, TNF-, and LPS-specific genes. Those genes whose expression was altered by more than one stimulus were not considered for the purpose of this paper.

### Development of the observed interactome of bladder mucosa and detrusor specific genes

The accession number and the ratio (treated/saline) for each mucosa- and detrusor-gene whose expression was altered by at least 3-fold by each stimuli were uploaded as focused genes in the Ingenuity Pathways Analysis [IPA], (Ingenuity Systems, Mountain View, CA), as described [69]. IPA is a robust and expertly curated database containing up-to-date information on over 20,000 mammalian genes and proteins, 1.4 million biological interactions, and one hundred canonical pathways incorporating over 6,000 discreet gene concepts. This information is integrated with other relevant databases such as EntrezGene and Gene Ontology [20,78]. IPA computes a score for each network according to the fit of the set of supplied focus genes (BCG-, TNF-, and LPS-dependent genes). These scores, derived from *p *values, indicate the likelihood of focus genes to belong to a network versus those obtained by chance. A score >2 indicates a ≥ 99% confidence that a focus gene network was not generated by chance alone [20]. Mathematically, the score is simply the negative exponent of the p-value calculation. For example, if the score is 3, then the corresponding p-value was 10^-3^, i.e. there is a 1 in 1000 chance that the Network Eligible Molecules found in that network appeared there just by chance. The experimental datasets of stimulus-dependent genes was used to query the IPA and to compose a set of interactive networks taking into consideration canonical pathways, the relevant biological interactions, and the cellular and disease processes.

### Chromatin immunoprecipitation (CHIP) quantitative real-time polymerase chain reaction (Q-PCR)-Based Assays

To determine whether intravesical BCG treatment would alter bladder cytokine gene expression, we used chromatin immunoprecipitation combined with Q-PCR. For this purpose, female C57BL/6J mice were anesthetized and instilled with 200 μl of one of the following substances: BCG (TheraCys^®^-Aventis-Pasteur; total dose of 1.35 mg) or pyrogen-free saline on days 1, 7, 14, and 21, as described above. Mice were euthanized with pentobarbital (200 mg/kg, i.p.) 24 hours after a single instillation (**BCG acute**) or 7 days after 4 weekly instillations (**BCG chronic**). A total of 60 mice were used (20 mice per group). Bladders were removed rapidly, frozen, and were shipped to Genpathway [79] for querying the chromatin for gene transcription (Genpathway's TranscriptionPath Query assay) [80].

The urinary bladders were exposed briefly to formaldehyde for cross-linking of the proteins and DNA together, followed by sonication to fragment the DNA into pieces of approximately 300–500 base pairs. An antibody against RNA polymerase II (Abcam) was then used to precipitate the DNA transcriptome. The Ab-protein-DNA complexes were purified using beads coupled to protein A. The DNA was isolated from the complexes using a combination of heat to reverse cross-linking, RNase and proteases, and then purified using phenol extraction and EtOH precipitation. The final CHIP DNAs were then used as templates in quantitative PCR reactions using primer pairs specific for each gene of interest. Quantitative PCR was carried out using Taq polymerase (iQ SYBR Green Supermix, Bio-Rad). Primer pairs were designed using Primer 3 [81]. Details of the primer sequences are given in Table S4 (Additional File 4). The designed primers shared 100% homology with the target sequence but no significant homology with other sequences.

Q-PCRs were run in triplicate and the values were transferred into copy numbers of DNA using a standard curve of genomic DNA with known copy numbers. The resulting transcription values for each gene were also normalized for primer pair amplification efficiency using the Q-PCR values obtained with Input DNA (unprecipitated genomic DNA). Results are presented as Transcription Events Detected Per 1000 Cells for each gene tested.

#### Statistical Analysis – Q-PCR

The difference between two mean values was analyzed with an unpaired Student's *t*-test (GraphPad Prism software version 4.0; GraphPad Software, Inc. San Diego, CA). A nominal *p *value less than 0.05 was considered statistically significant.

#### Electrophoretic mobility shift assays (EMSA)

Anesthetized C57BL6 female mice were instilled with 200 μl of one of the following substances: BCG (TheraCys^®^-Aventis-Pasteur; total dose of 1.35 mg) or pyrogen-free saline on days 1, 7, 14, and 21, as described above. Mice were euthanized with pentobarbital (200 mg/kg, i.p.) 24 hours after a single instillation (**BCG acute**) or 7 days after 4 weekly instillations (**BCG chronic**). Urinary bladders were placed in cold phosphate buffered saline (0°C), containing protease inhibitors (Protease Inhibitor Cocktail, Sigma, St. Louis, MO) and phosphatase inhibitors (20 mM sodium fluoride, 1 mM β-glycerophosphate, and 1 mM sodium orthovanadate).

The detrusor muscle presents a high level of complexity which makes difficult the interpretation of transcriptional factor binding results [22]. To decrease the complexity of using whole bladder homogenates, the bladder mucosa was separated from the detrusor smooth muscle, as described [22,68]. Briefly, immediately after removal from the animal, bladders were placed in PBS with protease inhibitors on ice and visualized under a dissecting microscope (Nikon SMZ 1500) and the detrusor smooth muscle was separated by blunt dissection away from the mucosa which contained the epithelium and sub-epithelial layers. Isolated layers were flash frozen and stored at -80°C until processing. Tissues were pulverized in a spring-loaded tissue pulverizer (Bio-Pulverizer, Biospec Products, Bartlesville, OK) chilled with liquid nitrogen. Nuclear and cytosolic extracts were prepared using the Pierce NE-PER Kit that enables stepwise separation and preparation of cytoplasmic and nuclear extracts from bladder tissue. Addition of the first two reagents (Pierce's proprietary information) to the pulverized tissue causes disruption of cell membranes and release of cytoplasmic contents. After recovering the intact nuclei from the cytoplasmic extract by centrifugation at 16,000 × g for 5 minutes, the nuclei are lysed with a third reagent (Pierce's proprietary information) to yield the nuclear extract. Extracts obtained with this product generally have less than 10% contamination between nuclear and cytoplasmic fractions-sufficient purity for most experiments involving nuclear extracts. A western blot was prepared using the nuclear and cytosolic extracts and probed for the nuclear proteins histone H3 and lamin A/C. No nuclear contamination was shown in the cytosolic fractions (data not shown). Protein concentrations were determined with a Micro BCA Kit (Pierce, Rockford, IL) per manufacturer's instructions.

#### NF-kappaB EMSA

A double-stranded NF-kappaB probe, 5' AGT TGA GGG GAC TTT CCC AGG 3' (Promega, Madison, WI), was end-labeled with [γ-^32^P] ATP (3000 Ci/mmole; GE Healthcare) and T4 polynucleotide kinase (New England Biolabs), and then purified using a G-50 column (GE Healthcare). Nuclear extracts (10 μg) were incubated with 2 ng of [^32^P] NF-kappaB double-stranded probe in a final volume of 20 μl containing 10 mM Tris HCl pH 8.0, 50 mM KCl, 1 mM DTT, 0.5 mM EDTA, 0.5% Triton X-100, 12.5% glycerol, and 1 μg poly d(I-C). For competition reactions, a 50-fold excess of unlabelled NF-kappaB probe was added to the reaction mixture, and it was incubated at room temperature for 10 minutes before the addition of the [^32^P] NF-kappaB double-stranded probe. Supershift reactions were pre-incubated for 20 minutes with antibody against p65 or p50 (p65, sc-109X, and p50, sc-114X, Santa Cruz Biotechnology, Santa Cruz, CA). Reaction mixtures were incubated for 30 minutes at room temperature. DNA-protein complexes were resolved on a non-denaturing 5% polyacrylamide gel at 1 mA/cm^2 ^2.5 hours in 0.5 TBE (45 mM Tris-borate and 1 mM EDTA). Gels were vacuum-dried and visualized on Kodak Biomax MS Film and quantified using ImageJ Software (NIH) and statistical differences were determined using GraphPad (version 4.0; GraphPad Software, Inc., San Diego, CA).

#### Target validation by Q-PCR of Chromatin Immunoprecipitation (CHIP)-Based Assays

Target validation was sought for genes known to have a binding site for NF-kappaB p65. For this purpose, the respective gene accession was uploaded into PAINT [Promoter Analysis and Interaction Network Toolset; [82]] to query the TRANSFAC database [83]. For target validation, human urothelial cancer cell line (J82) was challenged with saline or BCG for 24 hours, a p65 antibody (sc-109, rabbit polyclonal, Santa Cruz Biotehcnology) was used to precipitate the chromatin, and ChiP-Q-PCR was used to quantify the message for p65-dependent genes.

#### Human Urothelial Cell Culture

Human bladder carcinoma cell line J82 (HTB-1) was obtained from the American Tissue Culture Collection. J82 cells were seeded onto a 4-chamber slide and cultured in Minimum Essential Media (MEM) supplemented with 10% fetal bovine serum (FBS), 100 μM non-essential amino acids, 1 mM sodium pyruvate, 100 U/ml penicillin/streptomycin and 2× MEM Vitamin Solution. Cells were maintained at 37°C in a humidified atmosphere containing 5% CO2 until 90% confluence was reached. The medium was removed and cells were fixed in 4% formaldehyde.

#### Challenge of J82 with BCG and ChIP generation

For this purpose, cells were cultured in 75 cm^2 ^flasks (10 ml media/1 ml cells), starved overnight using MEM, and challenged for 24 hours with control saline or BCG (8.1 × 106 colony forming units [CFU]). Following 24 hours of challenge, 10^6 ^cells from both groups were frozen for ChIP-Q-PCR and shipped to Genpathway [79] for querying the chromatin for detection and quantification of p65 binding upstream of specific genes using chromatin immunopreciptation (ChIP) combined with real time PCR (Q-PCR) (Genpathway's FactorPath Query assay) [80]. J82 cells were exposed briefly to formaldehyde for cross-linking of the proteins and DNA together, followed by sonication to fragment the DNA into pieces of approximately 300–500 bp. A p65 antibody (sc-109, Santa Cruz Biotechnology) was then used to precipitate the p65-DNA complexes. The Ab-protein-DNA complexes were purified using beads coupled to protein G. The DNA was isolated from the complexes using a combination of heat to reverse cross-linking, RNase and proteases, and then purified using phenol extraction and EtOH precipitation. The final ChIP DNAs were then used as templates for Q-PCR reactions using primer pairs specific for each genomic region of interest. Q-PCR was carried out using Taq polymerase (iQ SYBR Green Supermix, Bio-Rad). Primer pairs were designed using Primer 3 [81]. Details of the primer sequences and the Genebank accession numbers are given in Table S4 (Additional File 4). The designed primers shared 100% homology with the target sequence but no significant homology with other sequences.

#### Q-PCR

Q-PCR reactions were run in triplicate and the averaged Ct values were transferred into copy numbers of DNA using a standard curve of genomic DNA with known copy numbers. The resulting binding values for each genomic region were also normalized for primer pair amplification efficiency using the Q-PCR values obtained with Input DNA (un-precipitated genomic DNA). Results are presented as binding events detected per 1000 cells for each genomic region tested and compared to an un-transcribed region used as a negative control. The difference between two mean values was analyzed with an unpaired Student's *t*-test (GraphPad Software, Inc., San Diego, CA). A nominal *p *value less than 0.05 was considered statistically significant.

#### Co-localization of BCG-GFP with LRG47 in the mouse bladder urothelium

Recombinant Mycobacterium bovis bacillus Calmette-Guerin expressing green fluorescent protein (rBCG-GFP), was constructed and provided by Dr. Michael O'Donnell [84,85]. rBCG-GFP was plated on Middlebrook 7H10 Bacto agar (Difco) supplemented with 10% ADC and 30 mg of kanamycin per ml. Individual colonies showing bright green fluorescence under illumination from a long-wavelength UV lamp were picked and grown in BCG culture medium for the internalization studies [84,85]. Cells were grown in flat-bottomed six-well tissue culture plates (Nunc) containing RPMI 1640 medium (Gibco BRL) supplemented with 10% heatinactivated fetal bovine serum and 30 mg of kanamycin per ml. When the cell density reached approximately 70% confluence, the monolayer cells were washed once with culture medium, and 1.5 × 10^7 ^CFU of GFP-expressing rBCG in 200 microliters were instilled into the bladder of anesthetized mice, as described above. Twenty-four hours after instillation, the bladders were removed and frozen for morphological analyses.

#### Morphology of the mouse urinary bladder secondary to rBCG-GFP instillation

Frozen bladders were processed for routine immunohistochemistry according to published methods [22,86]. Frozen sections were post-fixed in 1% formaldehyde. All reagent incubations and washes were performed at room temperature and routine H&E sections and immunofluorescence were performed. For the latter, 5% normal donkey blocking serum (Jackson Immunolabs) was placed on all slides for 45 min and sections were incubated with primary antibody for 1 hour and 45 minutes in a humidified chamber. Slides were washed 3 × 5 minutes in PBS and incubated with secondary antibodies. Slides were washed, counterstained with DAPI (1:20,000 dilutions of 10 mg/ml) for 2 minutes and coverslipped with Shur/Mount (TBS) mounting media and sealed with nail polish. All tissue sections were visualized using a Nikon inverted fluorescent microscope (Eclipse TE2000 coupled with epi-fluorescence attachment) [87] and imaged at room temperature using a FC9900 Cooled camera (Kodak chip: KAF3200ME w/microlens technology and 4-million pixels in a 2184 × 1472 array; F2.8 manual zoom lens and F1.4 fixed lens) driven by NIS-Elements AR2.3 Imaging software (Laboratory Imaging/Nikon) [88]. The LRG47 (A19) antibody (Santa Cruz Biotech) was used at 1:50 dilution. Secondary antibody, donkey anti-rabbit IgG AF488 conjugate (Molecular Probes; ), was used at 1:400 dilution. Control included omission of the primary antibody.

#### Image analyses

H&E sections were photographed at 200× and 400×. For composite H&E pictures, at total of 9 photos were taken at 200× and automatically merged in Adobe Photoshop^® ^CS3 extended [89]. For immunofluorescence, on the fluorescence microscope, the background fluorescence (nonspecific fluorescence of the tissue) of each image was set to a barely detectable level by adjusting the gain of the charge-coupled device camera, and then the image was captured. To determine the peak of fluorescence intensity, RGB images (BCG-GFP, and LRG47) were placed in the same canvas using Adobe Photoshop^® ^CS3 extended [89] and were analyzed using Image J software [23] using an Interactive 3D Surface Plot [90]. The Integrative 3D surface translates the luminance of an image as height for the plot. Internally, the image is scaled to a square image using nearest neighbor sampling, next the viewing position of the plot was adjusted for the nest visualization of the surface plot. For co-localization of BCG-GFP and LRG47-Rhodamine, pictures were converted to 8-bit gray scale images (fluorescence intensity range: 0 < 255) using Image J software [23] and the analysis was performed with the co-localization-finder plug-in [24]. The results are presented in an electronically merged picture with white areas indicative of co-localization and the results were automatically tabled with computer Pearson's correlation, overlap coefficient, contribution of both channels to overlap coefficient, slope and intercept of the linear regression, % pixels selected, min and max in both channels.

## Competing interests

The author(s) declare that they have no competing interests.

## Authors' contributions

MRS participated in its design, carried out the animal experiments, removed the tissues, extracted the RNA, and performed cDNA arrays; CS treated the animals with BCG and performed EMSAs; ID normalized the data and performed statistical analysis of gene-array results; CAD was responsible for the animal husbandry, photomicrographs, and image analysis; REH participated in the experimental design and helped drafting this manuscript; MAO consulted RS regarding clinical implications of BCG treatment, data interpretation, and helped drafting the manuscript; W-RW consulted RS regarding uroplakins, data interpretation, and helped drafting the manuscript; and RS conceived of the study, analyzed the gene-array together with ID, developed the IPA analysis and generated the pathways, and drafted the manuscript. All authors have read and approved the final manuscript.

## Supplementary Material

Additional file 1 Table S1. TNF-Specific Genes Click here for file

Additional file 2 Table S2. LPS-Specific Genes Click here for file

Additional file 3 Table S3. BCG-Specific Genes Click here for file

Additional file 4 Table S4. Mouse and Human Primers Click here for file
