# Supplementary material for: Molecular networks discriminating mouse bladder responses to intravesical bacillus Calmette-Guerin (BCG), LPS, and TNF-α
Source: BMC Immunol. 2008 Feb 11;9:4. doi: 10.1186/1471-2172-9-4 (PMC2262873; doi:10.1186/1471-2172-9-4)
Supplement: Additional file 1 — Table S1. TNF-Specific Genes [file 1471-2172-9-4-S1.pdf]

Table 1 TNF-specific genes

	ID	Genes	Description	Location	Type
TNF DETRUSOR	*ID*	*Genes*	*Description*	*Location*	*Type*
	AF010305	BHLHB2	basic helix-loop-helix domain containing, class B, 2	Nucleus	transcription regulator
	NM_016787	BNIP2	BCL2/adenovirus E1B 19kDa interacting protein 2	Cytoplasm	other
	NM_010796	CLEC10A	C-type lectin domain family 10, member A	Plasma Membrane	other
	NM_007875	DPAGT1	dolichyl-phosphate (UDP-N-acetylglucosamine)	Cytoplasm	enzyme
	L21671	EPS8	epidermal growth factor receptor pathway substrate 8	Plasma Membrane	peptidase
	NM_010356	GSTA3	glutathione S-transferase A3	Cytoplasm	enzyme
	S73496	LIFR	leukemia inhibitory factor receptor alpha	Plasma Membrane	transmembrane receptor
	U08354	MC5R	melanocortin 5 receptor	Plasma Membrane	G-protein coupled receptor
	NM_011014	OPRS1	opioid receptor, sigma 1	Plasma Membrane	G-protein coupled receptor
	NM_011057	PDGFB	platelet-derived growth factor beta polypeptide	Extracellular Space	growth factor
	NM_008883	PLXNA3	plexin A3	Plasma Membrane	transmembrane receptor
	NM_008856	PRKCH	protein kinase C, eta	Cytoplasm	kinase
	Z22819	RAB24	RAB24, member RAS oncogene family	Cytoplasm	enzyme
	NM_011235	RAD51L3	RAD51-like 3 (S. cerevisiae)	Nucleus	enzyme
	NM_009063	RGS5	regulator of G-protein signalling 5	Plasma Membrane	other
	U29056	SLA	Src-like-adaptor	Plasma Membrane	other
	NM_009288	STK10	serine/threonine kinase 10	Cytoplasm	kinase
TNF MUCOSA	*ID*	*Genes*	*Description*	*Location*	*Type*
	L07049	ACAN	aggrecan	Extracellular Space	other
	D12487	CHAT	choline acetyltransferase	Nucleus	enzyme
	NM_010338	GPR37	G protein-coupled receptor 37 (endothelin receptor type B-like)	Plasma Membrane	G-protein coupled receptor
	NM_010476	HSD17B7	hydroxysteroid (17-beta) dehydrogenase 7	Cytoplasm	enzyme
	NM_016692	INCENP	inner centromere protein antigens 135/155kDa	Nucleus	other
	NM_017404	MRPL39	mitochondrial ribosomal protein L39	Cytoplasm	other
	M12731	MYCN	v-myc myelocytomatosis viral related oncogene, neuroblastoma derived	Nucleus	transcription regulator
	NM_017373	NFIL3	nuclear factor, interleukin 3 regulated	Nucleus	transcription regulator
	NM_019955	RIPK3	receptor-interacting serine-threonine kinase 3	Plasma Membrane	kinase
	NM_011325	SCNN1B	sodium channel, nonvoltage-gated 1, beta	Plasma Membrane	ion channel
	NM_011709	WAP	whey acidic protein	Extracellular Space	other
