# Supplementary material for: Molecular networks discriminating mouse bladder responses to intravesical bacillus Calmette-Guerin (BCG), LPS, and TNF-α
Source: BMC Immunol. 2008 Feb 11;9:4. doi: 10.1186/1471-2172-9-4 (PMC2262873; doi:10.1186/1471-2172-9-4)
Supplement: Additional file 2 — Table S2. LPS-Specific Genes [file 1471-2172-9-4-S2.pdf]

Table 2 LPS-specific genes

	ID	Genes	Description	Location	Type
LPS DETRUSOR	*ID*	*Genes*	*Description*	*Location*	*Type*
	L41145	BMP5	bone morphogenetic protein 5	Extracellular Space	growth factor
	NM_009131	CLEC11A	C-type lectin domain family 11, member A	Extracellular Space	growth factor
	NM_009146	FRRS1	ferric-chelate reductase 1	Plasma Membrane	transmembrane receptor
	NM_008131	GLUL	glutamate-ammonia ligase (glutamine synthetase)	Cytoplasm	enzyme
	NM_010774	MBD4	methyl-CpG binding domain protein 4	Nucleus	enzyme
	NM_016682	SAE2	SUMO1 activating enzyme subunit 2	Cytoplasm	enzyme
	NM_019575	SCAMP4	secretory carrier membrane protein 4	Unknown	other
	NM_015749	TCN2	transcobalamin II; macrocytic anemia	Extracellular Space	transporter
	Y08975	UNG	uracil-DNA glycosylase	Nucleus	enzyme
	NM_009480	USF1	upstream transcription factor 1	Nucleus	transcription regulator
LPS	*ID*	*Genes*	*Description*	*Location*	*Type*
MUCOSA	NM_008339	CD79B	CD79b molecule, immunoglobulin-associated beta	Plasma Membrane	transmembrane receptor
	NM_019500	CLDN14	claudin 14	Plasma Membrane	other
	NM_009990	CLIP2	CAP-GLY domain containing linker protein 2	Cytoplasm	transcription regulator
	U55075	F2R	coagulation factor II (thrombin) receptor	Plasma Membrane	G-protein coupled receptor
	NM_008104	GCM2	glial cells missing homolog 2 (Drosophila)	Nucleus	transcription regulator
	NM_013569	KCNH2	potassium voltage-gated channel, subfamily H	Plasma Membrane	ion channel
	NM_019648	METAP2	methionyl aminopeptidase 2	Cytoplasm	peptidase
	NM_008605	MMP12	matrix metallopeptidase 12 (macrophage elastase)	Extracellular Space	peptidase
	NM_008731	NPY2R	neuropeptide Y receptor Y2	Plasma Membrane	G-protein coupled receptor
	NM_011850	NR0B2	nuclear receptor subfamily 0, group B, member 2	Nucleus	ligand-dependent nuclear receptor
	NM_008737	NRP1	neuropilin 1	Plasma Membrane	transmembrane receptor
	NM_020292	OLFR672	olfactory receptor 672	Plasma Membrane	G-protein coupled receptor
	NM_008860	PRKCZ	protein kinase C, zeta	Cytoplasm	kinase
	NM_011206	PTPN18	protein tyrosine phosphatase, non-receptor type 18 (brain-derived)	Nucleus	phosphatase
	NM_019713	RASSF1	Ras association (RalGDS/AF-6) domain family 1	Nucleus	other
	NM_009529	SLX	Sycp3 like X-linked	Nucleus	other
	NM_013679	SVS6	seminal vesicle secretory protein 6	Extracellular Space	other
	NM_019981	TEX101	testis expressed 101	Unknown	other
