# Supplementary material for: Molecular networks discriminating mouse bladder responses to intravesical bacillus Calmette-Guerin (BCG), LPS, and TNF-α
Source: BMC Immunol. 2008 Feb 11;9:4. doi: 10.1186/1471-2172-9-4 (PMC2262873; doi:10.1186/1471-2172-9-4)
Supplement: Additional file 3 — Table S3. BCG-Specific Genes [file 1471-2172-9-4-S3.pdf]

Table 3 BCG-specific genes

BCG	*ID*	*Genes*	*Description*	*Location*	*Type*
DETRUSOR	NM_019816	AATF	apoptosis antagonizing transcription factor	Nucleus	transcription regulator
	NM_007422	ADSS	adenylosuccinate synthase	Cytoplasm	enzyme
	NM_017402	ARHGEF7	Rho guanine nucleotide exchange factor (GEF) 7	Cytoplasm	other
	NM_007880	ARID3A	AT rich interactive domain 3A (BRIGHT-like)	Nucleus	transcription regulator
	NM_019665	ARL6	ADP-ribosylation factor-like 6	Cytoplasm	transporter
	NM_007490	ART2A	ADP-ribosyltransferase 2a	Extracellular Space	enzyme
	NM_011797	CA14	carbonic anhydrase XIV	Plasma Membrane	enzyme
	L02844	CD22	CD22 molecule	Plasma Membrane	other
	NM_010016	CD55	CD55 molecule, decay accelerating factor for complement	Plasma Membrane	other
	L25606	CD86	CD86 molecule	Plasma Membrane	transmembrane receptor
	AB008811	CDH2	cadherin 2, type 1, N-cadherin (neuronal)	Plasma Membrane	other
	NM_017478	COPG2	coatomer protein complex, subunit gamma 2	Cytoplasm	transporter
	NM_011991	COPS3	COP9 constitutive photomorphogenic homolog subunit 3	Cytoplasm	other
	U05247	CSK	c-src tyrosine kinase	Cytoplasm	kinase
	U90331	CTNND2	catenin (cadherin-associated protein), delta 2	Plasma Membrane	other
	NM_010046	DGAT1	diacylglycerol O-acyltransferase homolog 1	Cytoplasm	enzyme
	NM_016811	DGKA	diacylglycerol kinase, alpha 80kDa	Cytoplasm	kinase
	NM_007839	DHX15	DEAH (Asp-Glu-Ala-His) box polypeptide 15	Nucleus	enzyme
	NM_016775	DNAJC5	DnaJ (Hsp40) homolog, subfamily C, member 5	Plasma Membrane	other
	NM_019680	ELF4	E74-like factor 4 (ets domain transcription factor)	Nucleus	transcription regulator
	Z32815	ELK3	ELK3, ETS-domain protein (SRF accessory protein 2)	Nucleus	transcription regulator
	U72523	ENAH	enabled homolog (Drosophila)	Cytoplasm	other
	U39643	FAF1	Fas (TNFRSF6) associated factor 1	Nucleus	other
	NM_008033	FNTA	farnesyltransferase, CAAX box, alpha	Cytoplasm	enzyme
	NM_010259	GBP2 (include	guanylate binding protein 2, interferon-inducible	Cytoplasm	enzyme
	NM_008137	GNA14	guanine nucleotide binding protein (G protein), alpha 14	Plasma Membrane	enzyme
	NM_013531	GNB4	guanine nucleotide binding protein (G protein), beta polypeptide 4	Plasma Membrane	enzyme
	NM_010316	GNG3	guanine nucleotide binding protein (G protein), gamma 3	Plasma Membrane	enzyme
	NM_010382	HLA-DRB1	major histocompatibility complex, class II, DR beta 1	Plasma Membrane	transmembrane receptor
	NM_010467	HOXD1	homeobox D1	Nucleus	transcription regulator
	NM_010473	HRC	histidine rich calcium binding protein	Cytoplasm	other
	U39391	HTR1A	5-hydroxytryptamine (serotonin) receptor 1A	Plasma Membrane	G-protein coupled receptor
	NM_011772	IKZF4	IKAROS family zinc finger 4 (Eos)	Nucleus	transcription regulator
	M84746	IL9R	interleukin 9 receptor	Plasma Membrane	transmembrane receptor
	NM_00108296	ITGAM	integrin, alpha M (complement component 3 receptor 3 subunit)	Plasma Membrane	other
	NM_010596	KCNA7	potassium voltage-gated channel, shaker-related subfamily, member 7	Plasma Membrane	ion channel
	U65591	KCNAB1	potassium voltage-gated channel, shaker-related subfamily, beta member 1	Plasma Membrane	ion channel
	M64228	KCNB1	potassium voltage-gated channel, Shab-related subfamily, member 1	Plasma Membrane	ion channel
	NM_008467	KPNA4	karyopherin alpha 4 (importin alpha 3)	Nucleus	transporter
	NM_007947	LCN5	lipocalin 5	Cytoplasm	enzyme
	M17015	LTA	lymphotoxin alpha (TNF superfamily, member 1)	Extracellular Space	cytokine
	D13759	MAP3K8	mitogen-activated protein kinase kinase kinase 8	Cytoplasm	kinase
	NM_008590	MEST	mesoderm specific transcript homolog (mouse)	Extracellular Space	peptidase
	NM_011845	MID2	midline 2	Cytoplasm	other
	U43548	MLX	MAX-like protein X	Nucleus	transcription regulator
	NM_008609	MMP15	matrix metallopeptidase 15 (membrane-inserted)	Extracellular Space	peptidase
	X81143	MSH2	mutS homolog 2, colon cancer, nonpolyposis type 1 (E. coli)	Nucleus	enzyme
	NM_008657	MYF6	myogenic factor 6 (herculin)	Nucleus	transcription regulator
	X59060	MYF6	myogenic factor 6 (herculin)	Nucleus	transcription regulator
	L00923	MYO1B	myosin IB	Cytoplasm	other
	NM_019825	NCOA6	nuclear receptor coactivator 6	Nucleus	transcription regulator
	NM_011060	PADI3	peptidyl arginine deiminase, type III	Cytoplasm	enzyme
	NM_013784	PIGN	phosphatidylinositol glycan anchor biosynthesis, class N	Cytoplasm	enzyme
	NM_008845	PIP5K2A	phosphatidylinositol-4-phosphate 5-kinase, type II, alpha	Cytoplasm	kinase
	X62701	PLAUR	plasminogen activator, urokinase receptor	Plasma Membrane	transmembrane receptor
	NM_011104	PRKCE	protein kinase C, epsilon	Cytoplasm	kinase
	NM_008858	PRKD1	protein kinase D1	Cytoplasm	kinase
	NM_011172	PRODH	proline dehydrogenase (oxidase) 1	Cytoplasm	enzyme
	NM_011969	PSMA7	proteasome (prosome, macropain) subunit, alpha type, 7	Cytoplasm	peptidase
	NM_011214	PTPRU	protein tyrosine phosphatase, receptor type, U	Plasma Membrane	phosphatase
	NM_009017	RAET1B	retinoic acid early transcript beta	Plasma Membrane	other
	NM_009067	RALBP1	ralA binding protein 1	Cytoplasm	enzyme
	NM_009027	RASGRF2	Ras protein-specific guanine nucleotide-releasing factor 2	Cytoplasm	other
	NM_019570	REV1	REV1 homolog (S. cerevisiae)	Nucleus	enzyme
	NM_017395	RFX5	regulatory factor X, 5 (influences HLA class II expression)	Nucleus	transcription regulator
	NM_013876	RNF11	ring finger protein 11	Nucleus	other
	NM_019996	RNUXA	RNA U, small nuclear RNA export adaptor	Unknown	other
	NM_009071	ROCK1	Rho-associated, coiled-coil containing protein kinase 1	Cytoplasm	kinase
	NM_009113	S100A13	S100 calcium binding protein A13	Cytoplasm	other
	NM_016717	SCLY	selenocysteine lyase	Cytoplasm	enzyme
	L36179	SCN7A	sodium channel, voltage-gated, type VII, alpha	Plasma Membrane	ion channel
	NM_011345	SELE	selectin E (endothelial adhesion molecule 1)	Plasma Membrane	other
	X97817	SEMA5A	semaphorin 5A	Plasma Membrane	transmembrane receptor
	NM_013781	SH2D3C	SH2 domain containing 3C	Cytoplasm	other
	NM_009197	SLC16A2	solute carrier family 16, member 2	Plasma Membrane	transporter
	NM_011391	SLC16A7	solute carrier family 16, member 7	Plasma Membrane	transporter
	NM_011396	SLC22A5	solute carrier family 22, member 5	Plasma Membrane	transporter
	NM_009211	SMARCC1	SWI/SNF related, matrix associated	Nucleus	transcription regulator
	D49473	SOX17	SRY (sex determining region Y)-box 17	Nucleus	transcription regulator
	NM_011564	SRY	sex determining region Y	Nucleus	transcription regulator
	NM_019637	STYX	serine/threonine/tyrosine interacting protein	Cytoplasm	phosphatase
	NM_020264	SVS7	seminal vesicle secretory protein 7	Extracellular Space	other
	NM_008532	TACSTD1	tumor-associated calcium signal transducer 1	Plasma Membrane	other
	NM_011549	TFEB	transcription factor EB	Nucleus	transcription regulator
	NM_011599	TLE1	transducin-like enhancer of split 1	Nucleus	transcription regulator
	NM_011626	TMEM165	transmembrane protein 165	Plasma Membrane	other
	NM_009462	USP10	ubiquitin specific peptidase 10	Cytoplasm	peptidase
	NM_009500	VAV2	vav 2 oncogene	Extracellular Space	cytokine
	M84487	VCAM1	vascular cell adhesion molecule 1	Plasma Membrane	other
	NM_016873	WISP2	WNT1 inducible signaling pathway protein 2	Extracellular Space	growth factor
	NM_012017	ZNF346	zinc finger protein 346	Nucleus	other
	M98502	ZNF436	zinc finger protein 436	Nucleus	other
BCG	*ID*	*Genes*	*Description*	*Location*	*Type*
MUCOSA	NM_019774	AKAP8	A kinase (PRKA) anchor protein 8	Nucleus	other
	AF064071	APAF1	apoptotic peptidase activating factor 1	Cytoplasm	other
	Y16256	BSG	basigin (Ok blood group)	Plasma Membrane	other
	NM_020036	CALML5	calmodulin-like 5	Cytoplasm	other
	NM_016739	CAPRIN1	cell cycle associated protein 1	Plasma Membrane	other
	NM_009808	CASP12	caspase 12	Cytoplasm	peptidase
	NM_007886	DTNB	dystrobrevin, beta	Plasma Membrane	other
	NM_010064	DYNC1I2	dynein, cytoplasmic 1, intermediate chain 2	Cytoplasm	other
	U52951	EZH2	enhancer of zeste homolog 2 (Drosophila)	Nucleus	transcription regulator
	NM_013517	FCER2	Fc fragment of IgE, low affinity II, receptor for (CD23)	Plasma Membrane	other
	NM_019873	FKBPL	FK506 binding protein like	Unknown	other
	L36434	GNPTAB	N-acetylglucosamine-1-phosphate transferase, alpha and beta subunits	Unknown	enzyme
	NM_019691	GRIA4	glutamate receptor, ionotrophic, AMPA 4	Plasma Membrane	ion channel
	M13226	GZMA	granzyme A (granzyme 1, cytotoxic T-lymphocyte-associated serine esterase 3)	Cytoplasm	peptidase
	NM_013546	HEBP1	heme binding protein 1	Cytoplasm	other
	Z50013	HRAS	v-Ha-ras Harvey rat sarcoma viral oncogene homolog	Plasma Membrane	enzyme
	NM_008313	HTR4	5-hydroxytryptamine (serotonin) receptor 4	Plasma Membrane	G-protein coupled receptor
	X81581	IGFBP3	insulin-like growth factor binding protein 3	Extracellular Space	other
	NM_008324	INDO	indoleamine-pyrrole 2,3 dioxygenase	Cytoplasm	enzyme
	AB005141	KL	klotho	Extracellular Space	enzyme
	U69270	LDB1	LIM domain binding 1	Nucleus	transcription regulator
	NM_013590	LYZ	lysozyme (renal amyloidosis)	Extracellular Space	enzyme
	NM_008641	MAST2	microtubule associated serine/threonine kinase 2	Cytoplasm	kinase
	NM_013839	NR1H3	nuclear receptor subfamily 1, group H, member 3	Nucleus	ligand-dependent nuclear receptor
	NM_173440	NRIP1	nuclear receptor interacting protein 1	Nucleus	transcription regulator
	NM_008794	PCSK7	proprotein convertase subtilisin/kexin type 7	Cytoplasm	peptidase
	NM_019540	PFPL	pore forming protein-like	Unknown	other
	NM_019762	PKP3	plakophilin 3	Plasma Membrane	other
	NM_011121	PLK1	polo-like kinase 1 (Drosophila)	Nucleus	kinase
	L12687	PNMT	phenylethanolamine N-methyltransferase	Cytoplasm	enzyme
	NM_008911	PPOX	protoporphyrinogen oxidase	Cytoplasm	enzyme
	U03873	PRRX1	paired related homeobox 1	Nucleus	transcription regulator
	NM_011180	PSCD1	pleckstrin homology, Sec7 and coiled-coil domains 1(cytohesin 1)	Cytoplasm	other
	NM_011730	SLC6A18	solute carrier family 6, member 18	Plasma Membrane	transporter
	NM_008542	SMAD6	SMAD family member 6	Nucleus	transcription regulator
	NM_011467	SPR	sepiapterin reductase (7,8-dihydrobiopterin:NADP+ oxidoreductase)	Cytoplasm	enzyme
	NM_019576	THSD1	thrombospondin, type I, domain containing 1	Unknown	other
	NM_016792	TXNL1	thioredoxin-like 1	Cytoplasm	enzyme
	NM_009493	V2R4	vomeronasal 2, receptor, 4	Plasma Membrane	other
	NM_011776	ZP3	zona pellucida glycoprotein 3 (sperm receptor)	Extracellular Space	other
BCG	*ID*	*Genes*	*Description*	*Location*	*Type*
DETRUSOR	L23971	FMR1	fragile X mental retardation 1	Nucleus	other
AND	L34290	GNB5	guanine nucleotide binding protein (G protein), beta 5	Plasma Membrane	enzyme
MUCOSA	J03958	GSTA5	glutathione S-transferase A5	Cytoplasm	enzyme
	NM_019508	IL17B	interleukin 17B	Extracellular Space	cytokine
	M17015	LTA	lymphotoxin alpha (TNF superfamily, member 1)	Extracellular Space	cytokine
	NM_010745	LY86	lymphocyte antigen 86	Plasma Membrane	other
	NM_008976	PTPN14	protein tyrosine phosphatase, non-receptor type 14	Cytoplasm	phosphatase
	NM_011443	SOX2	SRY (sex determining region Y)-box 2	Nucleus	transcription regulator
	NM_009301	SVS5	seminal vesicle secretory protein 5	Extracellular Space	other
