# Supplementary material for: Molecular networks discriminating mouse bladder responses to intravesical bacillus Calmette-Guerin (BCG), LPS, and TNF-α
Source: BMC Immunol. 2008 Feb 11;9:4. doi: 10.1186/1471-2172-9-4 (PMC2262873; doi:10.1186/1471-2172-9-4)
Supplement: Additional file 4 — Table S4. Mouse and Human Primers [file 1471-2172-9-4-S4.pdf]

Table 4A Mouse Primers

PRIMER	*FORWARD*	*REVERSE*
ARL6	gcgctctcccaaaagtaata	gcaaaggacgtgaaggaaag
DNAJC5	gtgcatggtgctcacaggta	tttgctgctagtttgctcaca
CSK	gccctcaaagcacagatgtt	ggcccagctactcaggactt
FAF1	gctcaggccagtcttaattgtt	ctctgggcacaagtcctttag
FkbpI	ggagatggaggctatggagaa	gtcagcagcagtcggagag
GBP2	tgtgccccaatgaaaaataa	cagaggaggaggtcggattc
GZMA	agcccaaaagggtcaagact	tccctcaagaaagccacatt
HLA-DRB1	gggtttcagattttgtctgttt	caattaggaccacctcaaatca
Igfbp3	ccttccagcgttcctatcag	caccaacgtatcacccacag
IL17b	ctttccccactctcccagac	caaccaacccaacgccttac
INDO	ccgttagggtggaaatggtat	tggcccaagaagtagtctttc
MAP3K8	caggacccagagggacagta	tgacttcaccagccaaagtg
SELE	gatggcttgtgatggcttgt	gtttgacccaggaggatctg
SMAD6	agggaacagagtcggagtca	tgaggctgctccaggttaaa
TFEB	tgggctctgtggtaggtagg	ctagggaggggctcaatcag
VCAM1	tctagctccttcaccctgtg	ttgaatccagaaagctcctc
WISP2	gaggcttggagaggtgacag	cccagagttgctgcttgtc
Untr	tcaggcatgaaccaccatac	aacatccacacgtccagtga

Table 4B Human Primers used in the J82 cell line

PRIMER	*FORWARD*	*REVERSE*
Untr12	TGGACCTTTACCTGCTTTATCA	AGCAAGGACTAGGATGACAGAA
CXCL10	TGGGGCTAGTGTGCCATATT	AGCCAGCAGGTTTTGCTAAG
CXCL2	cgggagttacgcaagacag	atggttggggctggaaag
ICAM1	TGCAAGAAGATAGCCAACCA	acgagcaagtggcaaagatt
IL6	cctcaccctccaacaaagat	gcctcagacatctccagtcc
IL8	aatgaaaagatgagggtgcat	GCTTGTGTGCTCTGCTGTCT
